# Reorganizing territorial healthcare to avoid inappropriate ED visits: does the spread of Community Health Centres make Walk-in-Clinics redundant?

**DOI:** 10.1186/s12913-020-05648-x

**Published:** 2020-08-27

**Authors:** Cristina Ugolini, Anna Caterina Leucci, Lucia Nobilio, Gianfranco Bertè

**Affiliations:** 1 Department of Economics and CRIFSP-Advanced School for Health Policy, Alma Mater Studiorum University of Bologna, Bologna, Italy; 2 CRIFSP-Advanced School for Health Policy, Alma Mater Studiorum University of Bologna, Bologna, Italy; 3 ASSR-Regional Agency for Health and Social Care, Emilia-Romagna Region, Bologna, Italy; 4 Local Health Authority of Parma, Emilia-Romagna Region, Parma, Italy

**Keywords:** Walk-in-Clinics, Community Health Centres, Primary care, Inappropriate ED use, C21, I10, I18, H5

## Abstract

**Background:**

Community care has recently been restructured with the development of Community Health Centres (CHCs), forcing a general rethink on the survival of previous organizational solutions adopted to reduce inappropriate ED access, for example Walk-in-Clinics (WiCs).

**Methods:**

We focus on the Italian Emilia-Romagna Region that has made huge investments in CHC development, whilst failing to proceed at a uniform rate from area to area. Estimating panel count data models for the period 2015–2018, we pursue two goals. First we test the existence of a “CHC effect”, choosing five urban cities with different degree of development of the CHC model and assessing whether, all else being equal, patients treated by GPs who have their premises inside the CHC show a lower need to seek inappropriate care (Aim 1). Second, we focus our attention on Walk-in-Clinics, investigating the long-established WiC in the city of Parma that currently coexists with three CHCs recently established in the same catchment area. In this case we try to assess whether, and to what extent, the progressive development of the CHCs in the city of Parma has been affecting the dynamics of WiC access (Aim 2).

**Results:**

As regards Aim 1, we show that CHCs reduce the probability of inappropriate patient access to emergency care. As regards Aim 2, in the city of Parma patients whose GP belongs to the CHC are less likely to visit the WiC on a workday, with no significant change during the weekend when CHCs are closed, questioning the need to maintain them both in the same area when the CHC model is fully implemented.

**Conclusions:**

Our results confirm the hypothesis that expanding access to primary care settings diminishes inappropriate ED use. In addition, our findings suggest that where CHCs and WiCs coexist in the same area, it may be advisable to implement strategies that bring WiC activities into step with CHC-based general primary care reforms to avoid duplication.

## Background

In recent decades there has been increasing interest in improving primary care organization with specific emphasis, among other objectives, on reducing the number of ED referrals for avoidable non-severe conditions [1–3]. Aimed at reducing ED overuse –leading to needless expense, crowding and lower access to those in real need– wide-ranging organizational changes have been implemented in Italy mostly involving primary care, including initiatives designed to promote the extension of opening hours and out-of-hours care by GP groups [4–6], establish Walk-in-Clinics that are primary-care-based emergency services inside or near to hospitals [7, 8] or, more recently to a greater extent, establish Community Health Centres (CHCs, *Case della Salute* in Italian) able to provide a wider range of acute and chronic medical care and, by strengthening regional healthcare, useful in increasing the appropriateness of ED visits [9–11].

Although a common definition of these initiatives remains elusive (they are called in different countries Medical Homes, Patient-centered Medical Homes, Community Health Centres, etc.), CHCs’ critical component is a healthcare delivery practice that actively engages patients and provides them coordinated and comprehensive care by means of team-based care, evidence-based medicine, an integrated health information technology system, clinical decision support tools such as population-based registries and elements of the Chronic Care Model [9]. Developed in the United States during the Nineties under the name of Medical Home model to facilitate the management of patients with complex medical problems, these initiatives have been now transferred and implemented worldwide, for example in Canada [12, 13] as well in several European countries [11, 14]. In 2007 the Italian Ministry of Health identified the implementation of the Community Health Centre (CHC) model as a national priority for community healthcare [15] and since then several Italian Regions have been planning and implementing CHC projects, with a gradual dissemination of the model, depending on the amount of regional funding and the resources available at each local level.

Considered to be a promising cost-effective strategy for delivering better quality care especially to those with chronic diseases, multiple empirical studies mainly conducted for the United States have so far highlighted positive findings from these implementations, particularly in the area of lower inappropriate use of hospital services (both hospitalization and emergency department) [16–20], encouraging a general rethink of the previous organizational solutions adopted for this purpose.

In this paper we focus on the Emilia-Romagna Region that has recently made huge investments in the development of Community Health Centres as milestones of a more extensive health and social care reorganization process aimed at coping more properly with regional population needs. Focusing on inappropriate ED visits, our first goal is to investigate whether the CHCs are an organizational solution able to promote, among multiple other objectives, a more appropriate use of emergency services. Estimating panel count data models for the period 2015–2018, we test the existence of a “CHC effect”, choosing five urban cities with different degree of development of the CHC model and assessing whether, all else being equal, patients treated by GPs who have their premises inside the CHC receive better treatment, reducing the need for them to seek inappropriate care, compared to patients whose GP decides not to join the CHC or operates in areas where the CHC is not yet available. In the study we mark this investigation as our Aim 1.

As the development of the CHC model is an onerous process, in both organisational and financial terms, if we found evidence of the CHC’s ability to increase ED appropriateness, the NHS (National Health Service) would be forced to reconsider the advisability of leaving other existing organizational solutions unchanged in order to reduce duplication. To this end, in the second part of the paper we focus our attention on Walk-in-Clinics, fast track systems for minor injuries or illnesses in which community care is involved to contain the rising number of ED attenders presenting non-urgent health complaints [7, 8, 12, 21, 22]. Investigating the long-established WiC in the city of Parma that currently coexists with three CHCs recently established in the same catchment area, we try to assess whether, and to what extent, the progressive development of the CHCs in the city of Parma has been affecting the dynamics of WiC access. In the analysis, this investigation will be our Aim 2.

In a previous work referring to the period 2007–2010 [7] evidence was found that by extending the opening hours of GP practices it was already possible to lower WiC attendances, casting doubts on the expediency of letting both policies –WiC and longer GP opening hours– continue to coexist in the same context and with partially overlapping objectives. With the rapid development of the CHC-model designed for globally improving both scope and availability of primary care, these doubts return to the fore with the need to assess whether CHCs act as an alternative to WiCs in providing a primary-care-based emergency service.

## Methods

### Institutional details

Established in 1978, the Italian NHS introduced a Beveridge system based on universalism, comprehensiveness and equity. In the 1990s, a series of reforms led to the progressive regionalization of the NHS, giving regions political, administrative and financial responsibility for the organization and delivery of healthcare through the Local Health Authorities (LHAs) [23]. Primary care services are delivered by General Practitioners (GPs), independent NHS contractors, and are free of charge at the point of need. Registration with a family doctor is compulsory and each GP has a maximum of 1500 registered patients.

Emilia-Romagna is a Region located in the north east of Italy delivering healthcare to a population of approximately 4.4 million people through 8 LHAs. In 2010, the Regional Authority issued Resolution No. 291/2010 containing Regional Guidelines for Local Health Authorities (LHAs) to develop Community Health Centres (CHCs, Case della Salute in Italian) [24, 25]. Such implementation is considered a regional health priority as CHCs are managed by LHA Primary Care Departments and designed to become a benchmark for local communities in terms of improved integration between hospital and community services and between social and health services (thanks to the presence in their premises of social workers hired by the local municipality who in case of need work side by side with the healthcare staff), as well as managing chronic conditions that can be handled at a local level without resorting to hospital care. In particular, CHCs need to provide citizens with a well-defined unique access point to healthcare; organize, integrate and coordinate care and health communication to patients; strengthen the integration between hospitals and community care, also providing outpatient emergency healthcare management; develop diagnostic and integrated care pathways together with prevention programs targeting individuals, specific subgroups and the general population; manage chronic conditions through primary and specialist care integration [10].

Following Regional Authority approval of the CHC project in 2010, at year end 2018, 105 CHCs had been established (143 are scheduled by completion of the process), involving 484 GPs that decided to relocate within CHC facilities. This represents 16% of GPs operating in the Region (2908 in 2018). The number of operative CHCs increased from 42 in 2011 to 49 in 2012, to 55 in 2013 and 63 in 2014, 67 in 2015, 84 in 2016 and 105 in 2018. Across the 8 regional LHAs, the highest share of CHCs is in Romagna (31%), Parma (17%) and Bologna (15%) LHAs. At the end of 2018, the percentage of GPs joining their local CHC by relocating within CHC facilities amounts to 16.5% [26].

Whilst CHCs are developing at a rapid pace across the Region, the entire regional area’s only experience of a Walk-in-Clinic regards the one established in Parma back in the early 2000s [27]. The Parma LHA is located in the north-western area of the region and organized in four Health Districts, the largest one being the Parma District that covers the entire city of Parma and delivers primary care to approximately 223,000 people through GPs mostly operating in group practices. Since the early 2000s, the Parma LHA has encouraged GPs to form group practices and gradually reorganized their opening times to allow patients greater accessibility to primary care. At the same time, in order to address the rising number of inappropriate ED visits, in 2003 Parma LHA launched a Walk-in-Clinic close to the Parma Teaching hospital that resembles similar English and Canadian experiences [12, 21, 22], except for the fact that it is staffed by primary care or deputized physicians who are not part of the hospital staff but hired direct by the LHA. As Italian LHAs receive capitation payments and the teaching hospital is financed by an inpatient prospective payment system based on Diagnosis Related Groups (DRGs), Parma LHA’s decision to open a WiC inside the Parma Teaching hospital itself reflects the quest for a more competitive alternative to potentially inappropriate ED referral.

The clinic is open daily from 8 a.m.-8 p.m., 365 days a year, for the treatment of minor injuries and illnesses that cannot be put off for 24 to 48 h. Healthcare is provided by a team of seven clinicians supported by four nurses, with at least one physician and one nurse per 6-h shift. In addition, an emergency eye service run entirely by hospital specialists is co-located in the WiC. Patients can directly access the WiC free of charge, either instead of visiting their GP or when the GP is not at the surgery or when the ED front desk redirects the patient to the WiC after triaging as non-urgent (“triage out”). Admission to the Parma teaching hospital for further specialist investigation is always allowed should the clinician diagnose an acute and urgent illness.

The recent introduction of the CHC model has also involved the Parma LHA where 17 CHCs have been opened (29 CHCs are scheduled by completion of the process). As regards the city centre, 3 CHCs have been set up, the first in July 2013 (Parma Centro), the second in December 2014 (Pintor) and the third in January 2015 (Montanara), all operating in the municipal district having the same catchment area as the WiC. Geographical distance does not create a real barrier to accessibility to the different facilities as all three CHCs are located no more than 10 min from the WiC and the ED with good/excellent public transport services.

The introduction and expected ongoing dissemination of the CHC model could call into question the continuing existence of the WiC –at least in its current embodiment– if and only if the CHCs prove themselves capable, among other objectives, of producing more appropriate use of emergency services.

### Data

Data used in this research was taken from the Regional Healthcare Information System which includes detailed information on GPs and the use of healthcare services by all regional patients with the latter as our unit of observation. In addition, datasets of patient attendance at the Parma WiC are also available. The observation period runs from 2015 to 2018.

The patient is our unit of observation. As our dependent count variable for Aim 1, we focus on inappropriate ED utilization for approximately half a million residents over 18 years living in the urban areas of Bologna, Modena, Reggio Emilia, Ferrara and Parma, followed each year from 2015 to 2018, whose GP has or has not joined the local CHC, for a total number of 2,184,066 patient-years registered with a GP located in the 5 urban city centres. We have already mentioned the 3 Parma CHCs, whilst as regards the other city centres, Bologna has 3 CHCs that were opened in 2013 (San Vitale-San Donato), 2015 (Borgo-Reno) and 2017 (Navile). Reggio Emilia has 3 CHCs, one opened in 2015 (Nord) and two in 2016 (Ovest and Spallanzani). Ferrara has 2 CHCs opened in 2011 (Pontelagoscuro) and 2014 (Cittadella San Rocco), whereas the centre of Modena has none at all. To select inappropriate ED visits, we follow the Italian four-level triage system where white code visits represent the lowest priority and are considered by the Emiia-Romagna Regional Department of Health as strictly inappropriate [6], in particular those recorded during workdays from 8 a.m. to 8 p.m., when there is the possibility for patients to get in touch with their GP.

For Aim 2 we consider three outcome variables for the city of Parma: the number of WiC attendances from Monday to Friday from 8 a.m.-8 p.m. (the same opening hours considered when counting inappropriate ED visits), the number of WiC attendances during the weekend when local CHCs are closed and the number of WiC eye emergency treatments provided by the hospital eye ED co-located in the WiC.

### Statistical analysis

The outcome variables used in this research were integer count variables that can be modeled by Poisson regression and its several generalizations. Overdispersion and zero inflation were assessed for each outcome variable. We recall that overdispersion occurs when the data present more variability than that expected under the assumed (Poisson) distribution, causing the underestimation of the estimated standard errors and imposing the need of specific adjustments, whereas zero inflation refers to a situation of frequent zero-valued observations.

Given the characteristics of our data, we do not have to deal with overdispersion measured as the deviance or Pearson’s chi-square divided by the degrees of freedom. As for all our count-dependent variables this quantity is equal to 1 the conditional variance of the dependent variable is equal to the conditional mean, signaling that our data are not over dispersed and that Poisson is the first choice for modelling [28]. Our data are characterised by a large proportion of observations that did not report any inappropriate ED visits (97.7%) or any WiC attendances (96%). The Vuong statistics with values greater than 1.96 revealed the need to utilize zero inflation corrections for each dependent variable and to estimate a Zero-inflated Poisson specification (ZIP) [29, 30]. To compare ZIP with the standard Poisson, we used Akaike Information Criterion (AIC) [31] and Bayesian Information Criterion (BIC) [32], two probabilistic statistical tests that attempt to quantify both the performance and the complexity of the model. A lower AIC or BIC value indicates a better fit. According to the AIC and BIC criteria, the model with the lowest values of such criteria was always ZIP, confirming its best fit.

The basic idea under zero-inflated models is that excess zeros are generated by a separate data generating process apart from the counting process. Excess zeros can be modelled independently, thus this kind of model is composed of two parts: 1) a Poisson count model; 2) a logit model called the inflation model for predicting excess zeros. These two parts correspond ideally to two patient groups: one group whose counts are generated by the standard Poisson regression model and another group (called the absolute zero group) who have zero probability of a count greater than 0. Even though all statistics confirm that ZIP fits our data better, we have no strong theoretical reasons for postulating a two-class model [33]. In fact, the logit part of the ZIP model predicts the outcome of zero observations and, thus, indicates the likelihood of being in the group of patients that never attend the ED inappropriately or never visit the local WiC but we do not have a subgroup of patients who have from the beginning a zero probability of attending ED or WiC, in other words all patients have some non-zero risk of an avoidable visit. For this reason, we prefer to estimate both models, showing the standard Poisson model next to the ZIP model. To assess the programme’s impact, we present for each covariate its Incidence Rate Ratio (IRR) that compares the ratio of incidence between the exposed and the unexposed groups (when the IRR is close to 1 there is no difference in risk between the two groups, the IRR > 1 (< 1) suggests an increased (reduced) risk in the exposed group for that outcome). All the estimates are significant at the 5% level.

To pursue Aim 1, we estimated the above described models using data referred to the pooled sample of the 5 cities. Subsequently, to pursue Aim 2, we estimated again the same models focusing on the city of Parma. Statistical analyses were performed in SAS (version 9.4, SAS Institute Inc., Cary, NC).

## Results

Table 1 provides the descriptive statistics of our population, considering separately the pooled sample of the 5 cities and the city of Parma. Patients’ characteristics include age, gender, citizenship, presence of chronic diseases (diabetes, asthma, hypertension, coronary artery disease, chronic obstructive pulmonary disease, congestive heart failure) and enrolment with a GP that has moved his or her premise inside the local CHC, keeping the first year of membership distinct. 53% of our patients are female, 63% are over 50 years old and 55% have at least one chronic disease. Foreigners account for 2% of the total, while 10% of the sample have a GP who belongs to the local CHC, this figure doubling in the city of Parma where GP local CHC membership rises to 20%.

**Table 1:** Descriptive statistics

	5 cities		Parma	
	No.	%	N	%
No. Observations	1,984,320	100.00	351,866	17.73
Year				
2015	522,372	26.32	91,092	25.89
2016	517,802	26.09	90,064	25.6
2017	507,394	25.57	87,789	24.95
2018	436,752	22.01	82,921	23.57
Patient_age				
18–34	286,360	14.43	52,740	14.99
35–49	441,647	22.26	82,664	23.49
50–64	544,699	27.45	97,791	27.79
65–84	579,943	29.23	98,660	28.04
> = 85	131,671	6.64	20,013	5.69
Patient_gender				
Female	1,056,495	53.24	187,045	53.16
Male	927,825	46.76	164,821	46.84
Patient_citizenship				
Foreign	47,111	2.37	8458	2.4
Italian	1,937,209	97.63	343,408	97.6
Patient_chronic diseases				
No chronic disease	886,568	44.68	157,563	44.78
One chronic disease	452,153	22.79	83,182	23.64
Two chronic diseases	295,970	14.92	51,187	14.55
Three or more chronic diseases	349,629	17.62	59,934	17.03
Patient whose GP is first year in CHC				
No	1,964,357	98.99	346,885	98.58
Yes	19,963	1.01	4981	1.42
Patient whose GP is in CHC				
No	1,804,343	90.93	286,391	81.39
Yes	179,977	9.07	65,475	18.61

Table 2 summarises ED and WiC attendance for the period 2015–2018 representing the outcomes considered as our dependent count variables for Aim 1 and for Aim 2 respectively.

**Table 2:** ED and WiC visits, years 2015–2018

**ED resident visits, five cities**											
	**2015**	**2016**	**2017**	**2018**	**Total**	**GP not in CHC**	**GP in CHC**	**Total**			
**0**											
**N**	510,354	505,837	495,561	426,272	1,938,024	1,760,580	177,444	1,938,024			
**%**	97.7	97.69	97.67	97.6	97.67	97.57	98.59	97.66			
**1**											
**N**	10,943	10,796	10,655	9488	41,882	39,565	2317	41,882			
**%**	2.09	2.08	2.1	2.17	2.11	2.19	1.29	2.11			
**> = 2**											
**N**	1075	1168	1177	991	4411	4195	216	4411			
**%**	0.21	0.23	0.23	0.23	0.22	0.23	0.12	0.22			
**Total**											
	522,372	517,801	507,393	436,751	1,984,317	1,804,340	179,977	1,984,317			
						90.93	9.07	100			
WiC resident visits, Parma (excluding eye visits)											
	**2015**	**2016**	**2017**	**2018**	**Total**	**Total weekdays**	**Total week-end**	**Total eye visits**	**GP not in CHC**	**GP in CHC**	**Total**
0											
N	87,676	86,788	84,179	78,976	337,619	346,122	342,469	343,674	274,711	62,908	337,619
%	96.25	96.36	95.89	95.24	95.95	98.37	97.3291	97.67	95.92	96.08	
1											
N	2959	2810	3093	3325	12,187	5252	8244	7261	9995	2192	12,187
%	3.25	3.12	3.52	4.01	3.46	1.49	2.34	2.06	3.49	3.35	
> = 2											
N	567	624	697	794	2682	492	1154	932	1686	375	2061
%	0.01	0.01	0.01	0.01	0.76	0.14	0.33	0.26	0.59	0.57	
Total											
N	91,092	90,065	87,789	82,921	351,867	351,867	351,867	351,867	286,392	65,475	351,867
									81.39	18.61	100

The number of inappropriate ED visits over the period is quite stable amounting to more than 2% of the observation sample for a total of 46,293 visits, with a lower percentage of access for patients whose GP belongs to the local CHC (1.4% versus 2.4%).

In the second part of Table 2 we focus on Parma city centre residents producing a total of 351,867 patient-years. The visits to the local WiC regard 4% of our sample, with a higher influx during the weekend and for emergency eye visits, with a lower percentage of access for patients whose GP belongs to the local CHC (3.9% versus 4.1%). For the Parma WiC, the yearly number of visits is constantly around 23,000. In the period 2015–2018 the most common diagnosis was HEENT (head, eye, ear, nose, throat) with eye problems accounting for 39% of cases. The remaining less frequent diagnoses included orthopaedical, urological and gynaecological problems (4%), skin problems (3%) (data not shown but available on request).

### Aim 1: do the CHCs promote a more appropriate use of EDs?

Table 3 shows results of the estimated number of inappropriate ED visits considering the pooled analysis of the five city centres.

**Table 3:** ED inappropriate visits, five cities, 2015–2018

	Zero inflated Poisson Model										Standard Poisson				
	Zero inflated part					Poisson part									
	Estimate	95% CI		***p***-value	IRR	Estimate	95% CI		***p***-value	IRR	Estimate	95% CI		***p***-value	IRR
**Intercept**	1.73	1.61	1.85	<.0001		−1.87	−1.98	−1.77	<.0001		−3.77	−3.80	−3.74	<.0001	
**Patient age 35–49**	0.08	−0.04	0.20	0.20	1.08	0.00	−0.10	0.11	0.96	1.00	− 0.06	−0.09	− 0.03	<.0001	0.94
**Patient age 50–64**	0.22	0.10	0.34	0.00	1.24	− 0.04	−0.14	0.06	0.45	0.96	−0.23	− 0.26	− 0.20	<.0001	0.80
**Patient age 65–84**	0.40	0.28	0.52	<.0001	1.50	0.17	0.07	0.28	0.00	1.19	−0.17	− 0.21	− 0.14	<.0001	0.84
**Patient age > =85**	0.85	0.71	0.99	<.0001	2.34	0.26	0.13	0.39	0.00	1.30	−0.48	− 0.53	− 0.44	<.0001	0.62
**Female patient**	−0.12	− 0.17	− 0.06	<.0001	0.89	0.02	−0.03	0.07	0.38	1.02	0.12	0.10	0.14	<.0001	1.12
**Foreign patient**	−0.15	−0.32	0.01	0.07	0.86	0.24	0.10	0.39	0.00	1.27	0.37	0.32	0.42	<.0001	1.44
**Patient with 3 or more chronic diseases**	−0.21	−0.30	− 0.12	<.0001	0.81	0.63	0.55	0.71	<.0001	1.88	0.81	0.78	0.84	<.0001	2.25
**Patient with 2 chronic disease**	−0.16	− 0.26	− 0.07	0.00	0.85	0.38	0.30	0.47	<.0001	1.47	0.52	0.49	0.55	<.0001	1.69
**Patient with 1 chronic disease**	−0.15	− 0.24	− 0.06	0.00	0.86	0.23	0.15	0.31	<.0001	1.26	0.35	0.33	0.38	<.0001	1.43
**2016**	0.05	−0.03	0.13	0.19	1.05	0.06	−0.01	0.14	0.08	1.07	0.02	0.00	0.05	0.07	1.02
**2017**	0.08	0.01	0.16	0.03	1.09	0.11	0.04	0.18	0.00	1.11	0.04	0.01	0.06	0.00	1.04
**2018**	−0.02	− 0.10	0.06	0.59	0.98	0.06	−0.01	0.13	0.11	1.06	0.08	0.06	0.11	<.0001	1.09
**Patient whose GP is first year in CHC**	0.35	0.00	0.71	0.05	1.42	0.01	−0.33	0.34	0.96	1.01	−0.32	− 0.43	− 0.22	<.0001	0.73
**Patient whose GP is in CHC**	0.42	0.30	0.55	<.0001	1.53	−0.01	− 0.13	0.12	0.01	0.99	−0.40	−0.43	− 0.36	<.0001	0.67
**Patient with a local WiC**	0.28	0.07	0.49	0.01	1.32	−1.03	−1.22	−0.84	<.0001	0.36	−1.28	−1.32	−1.24	<.0001	0.28
**AICC**	466,453.14										480,644.32				
**BIC**	466,853.17										480,844.34				
**Pearson Chi-Square (value/DF)**	1.05										1.07				
**Vuong statistic**	5.41														

According to the results of the standard Poisson model (second part of Table 3), there is a negative and significant association between the availability of CHCs and WiCs and the probability of attending the ED inappropriately. For patients whose GP belongs to the local CHC, the magnitude of the reduction in inappropriate ED use is estimated at about 33% (IRR 0.67–95% IC: 0.65–0.69), whilst slightly lower in the first year (27%), with an IRR of 0.73 (95% IC: 0.65–0.80), whereas for patients living in an area with a local WiC, the estimated reduction is 72% (IRR 0.28–95% IC: 0.27–0.29). Inappropriate ED visits are in most cases negatively correlated with age, where the proportion of patients aged 18–34 is the one associated with the significantly higher inappropriate use of ED, and positively correlated with the proportion of females, foreigners and the level of chronicity.

As the results of the logit and Poisson parts of the ZIP model differ from each other (first part of Table 3) it can be argued that the factors affecting the decision not to attend ED inappropriately are different from the factors affecting the decision as to how much to use this service inappropriately. The probability of belonging to the absolute zero group increases with patient’s age, but diminishes for females, foreigners and patients with a growing number of chronic conditions. Interestingly, patients whose GP belongs to the local CHC have a higher probability of never attending the ED inappropriately (OR 1.53–95% CI:1.35–1.73); the same result holds for patients having a WiC local to them, though with minor intensity. In the Poisson part of the model, older patients, patients with more severe chronic conditions and foreigners have a higher probability of attending ED. Local WiC availability reduces the probability of increasingly attending ED by 64%, whereas CHC impact is modest, reducing the probability of attending ED repeatedly by just 1%. These findings suggest that the CHC impact is stronger in influencing the decision to avoid inappropriate ED use completely but it is a weaker factor when it comes to modifying the behaviour of the so-called frequent flyers.

### Aim 2: does the progressive development of the CHCs affect the dynamics of WiC access?

Table 4 presents the analysis for Parma city centre where the only WiC available in the Emilia-Romagna Region was established, considering all weekday access for any reason with the exception of eye visits that are managed directly by hospital specialists. In the standard Poisson model (second part of Table 4) the CHC impact on the probability of attending the local WiC is significant: patients whose GP belongs to the CHC have a lower 6% probability of attending the WiC (with no significant effect during the first year of membership (IRR 0.74–95% IC: 0.82–1.15). WiC visits are negatively correlated with age, positively correlated with the proportion of foreigners and patients with a more severe level of chronicity. In the zero-inflated part of ZIP estimation (first part of Table 4), the probability of belonging to the absolute zero group increases with patient’s age but diminishes for females and for chronic patients. The CHC has no significant impact on the probability of belonging to the absolute zero group whereas the effect is particularly strong in the Poisson part of the ZIP model where having a GP belonging to the CHC reduces the probability of weekday local WiC attendance by 11% (IRR 0.89–95% IC: 0.81–0.99), with a far more significant impact (43%) during the year the GP first joins (IRR 0.57–95% IC: 0.34–0.98) for reasons that would probably deserve a further investigation, preferably based on a questionnaire study. Older patients and patients with more severe chronic conditions have a higher probability of attending the WiC more than once but female patients are 9% less likely to increase their use of the WiC.

**Table 4:** WiC resident visits, Parma city centre, 2015–2018 (all visits, with the exclusion of eye visits), weekdays attendances

	Zero inflated Poisson Model										Standard Poisson				
	Zero inflated part					Poisson part									
	Estimate	95% CI		***p***-value	OR	Estimate	95% CI		***p***-value	IRR	Estimate	95% CI		***p***-value	IRR
**Intercept**	1.83	1.65	2.01	<.0001		−1.69	−1.86	−1.53	<.0001		−3.65	−3.71	−3.58	<.0001	
**Patient age 35–49**	0.26	0.09	0.44	0.00	1.30	0.09	−0.07	0.26	0.26	1.10	−0.14	−0.20	−0.08	<.0001	0.87
**Patient age 50–64**	0.55	0.38	0.73	<.0001	1.74	0.12	−0.04	0.28	0.15	1.13	−0.38	−0.45	− 0.32	<.0001	0.68
**Patient age 65–84**	0.96	0.78	1.13	<.0001	2.60	0.50	0.34	0.67	<.0001	1.65	−0.36	−0.43	−0.29	<.0001	0.70
**Patient age > =85**	1.67	1.46	1.87	<.0001	5.29	1.07	0.88	1.26	<.0001	2.92	−0.45	−0.55	− 0.35	<.0001	0.64
**Female patient**	− 0.11	− 0.20	− 0.03	0.01	0.89	− 0.10	− 0.18	− 0.02	0.01	0.91	− 0.02	− 0.06	0.02	0.28	0.98
**Foreign patient**	−0.12	− 0.40	0.17	0.42	0.89	0.09	−0.18	0.35	0.52	1.09	0.20	0.08	0.32	0.00	1.22
**Patient with 3 or more chronic diseases**	−0.37	− 0.51	− 0.24	<.0001	0.69	0.48	0.35	0.61	<.0001	1.61	0.85	0.79	0.91	<.0001	2.33
**Patient with 2 chronic disease**	−0.31	− 0.45	− 0.17	<.0001	0.73	0.30	0.16	0.43	<.0001	1.35	0.61	0.54	0.67	<.0001	1.83
**Patient with 1 chronic disease**	−0.35	− 0.49	− 0.22	<.0001	0.70	0.06	−0.07	0.18	0.39	1.06	0.39	0.33	0.44	<.0001	1.47
**2016**	0.59	0.45	0.72	<.0001	1.80	0.64	0.51	0.77	<.0001	1.89	0.12	0.06	0.17	<.0001	1.12
**2017**	0.43	0.29	0.56	<.0001	1.53	0.61	0.48	0.74	<.0001	1.84	0.25	0.19	0.30	<.0001	1.28
**2018**	0.01	−0.12	0.15	0.84	1.01	0.40	0.27	0.53	<.0001	1.49	0.41	0.35	0.46	<.0001	1.50
**Patient whose GP is first year in CHC**	−0.60	−1.23	0.02	0.06	0.55	−0.56	−1.09	− 0.02	0.04	0.57	−0.03	−0.20	0.14	0.74	0.97
**Patient whose GP is in CHC**	−0.05	− 0.16	0.05	0.33	0.95	−0.11	− 0.21	− 0.01	0.03	0.89	−0.06	− 0.11	− 0.01	0.01	0.94
**AICC**	98,489.93										106,945.10				
**BIC**	98,813.06										107,106.66				
**Pearson Chi-Square (value/DF)**	1.03										1.31				
**Vuong statistic**	4.89														

As natural robustness checks, we examine the impact on the probability of attending WiC during the weekend when CHCs are closed (Table 5) and for reasons concerning eye emergencies that are dealt with by the eye ED co-located in the WiC (Table 6). Both lend further support to our results, showing no significant effect for patients whose GP belongs to the local CHC when the CHC is closed or for reasons that are rarely managed by primary care.

**Table 5:** WiC resident visits, Parma city centre, 2015–2018 (all visits, with the exclusion of eye visits), week-end attendances

	Zero inflated Poisson Model										Standard Poisson				
	Zero inflated part					Poisson part									
	Estimate	95% CI		***p***-value	IRR	Estimate	95% CI		***p***-value	IRR	Estimate	95% CI		***p***-value	IRR
**Intercept**	2.03	1.72	2.33	<.0001		−1.94	−2.22	−1.66	<.0001		−4.08	−4.16	−3.99	<.0001	
**Patient age 35–49**	0.06	−0.25	0.36	0.71	1.06	−0.07	− 0.35	0.21	0.62	0.93	−0.12	− 0.20	− 0.04	0.00	0.89
**Patient age 50–64**	0.35	0.05	0.65	0.02	1.41	−0.06	− 0.34	0.22	0.67	0.94	−0.38	−0.46	− 0.29	<.0001	0.69
**Patient age 65–84**	0.53	0.21	0.86	0.00	1.70	−0.19	− 0.50	0.11	0.21	0.82	−0.68	−0.77	− 0.59	<.0001	0.50
**Patient age > =85**	1.43	1.00	1.87	<.0001	4.20	0.20	−0.23	0.62	0.36	1.22	−1.15	− 1.31	− 1.00	<.0001	0.32
**Female patient**	−0.05	− 0.23	0.12	0.55	0.95	0.04	−0.12	0.20	0.64	1.04	0.08	0.03	0.13	0.00	1.09
**Foreign patient**	0.19	−0.25	0.64	0.40	1.21	0.34	− 0.08	0.76	0.11	1.40	0.17	0.02	0.32	0.02	1.19
**Patient with 3 or more chronic diseases**	0.00	−0.27	0.27	0.99	1.00	0.78	0.52	1.04	<.0001	2.18	0.79	0.71	0.88	<.0001	2.21
**Patient with 2 chronic disease**	0.07	−0.20	0.34	0.60	1.07	0.68	0.43	0.94	<.0001	1.98	0.63	0.55	0.71	<.0001	1.88
**Patient with 1 chronic disease**	−0.02	− 0.27	0.23	0.89	0.98	0.36	0.13	0.59	0.00	1.43	0.38	0.31	0.45	<.0001	1.47
**2016**	0.09	−0.14	0.33	0.45	1.09	0.09	−0.14	0.31	0.45	1.09	0.00	−0.07	0.07	0.98	1.00
**2017**	−0.28	− 0.53	− 0.03	0.03	0.76	− 0.15	− 0.38	0.08	0.20	0.86	0.10	0.02	0.17	0.01	1.10
**2018**	−0.18	− 0.41	0.06	0.15	0.84	0.00	−0.22	0.22	1.00	1.00	0.16	0.09	0.23	<.0001	1.17
**Patient whose GP is first year in CHC**	−0.05	−0.64	0.54	0.87	0.95	0.17	−0.38	0.73	0.54	1.19	0.23	0.03	0.43	0.06	1.26
**Patient whose GP is in CHC**	−0.01	− 0.24	0.21	0.90	0.99	−0.05	−0.26	0.16	0.65	0.95	−0.04	−0.10	0.03	0.29	0.97
**AICC**	62,144.99										64,050.41				
**BIC**	62,468.11										64,211.97				
**Pearson Chi-Square (value/DF)**	1.02										1.03				
**Vuong statistic**	12.62

**Table 6:** WiC resident visits. Parma city centre. 2015–2018, **only eye visits**

	Zero inflated Poisson Model										Standard Poisson				
	Zero inflated part					Poisson part									
	Estimate	95% CI		***p***-value	IRR	Estimate	95% CI		***p***-value	IRR	Estimate	95% CI		***p***-value	IRR
**Intercept**	2.16	1.88	2.44	<.0001		−1.66	−1.92	−1.40	<.0001		−3.90	−3.98	−3.83	<.0001	
**Patient age 35–49**	0.12	−0.16	0.41	0.40	1.13	0.32	0.06	0.59	0.02	1.38	0.22	0.14	0.30	<.0001	1.24
**Patient age 50–64**	0.00	−0.28	0.29	0.97	1.00	0.20	−0.07	0.46	0.14	1.22	0.20	0.12	0.28	<.0001	1.22
**Patient age 65–84**	0.12	−0.17	0.41	0.42	1.13	0.41	0.14	0.68	0.00	1.51	0.31	0.23	0.40	<.0001	1.37
**Patient age > =85**	0.56	0.19	0.92	0.00	1.74	0.37	0.03	0.71	0.03	1.45	−0.13	−0.25	−0.01	0.04	0.88
**Female patient**	−0.16	−0.28	− 0.04	0.01	0.85	−0.24	− 0.35	−0.12	<.0001	0.79	−0.10	−0.14	− 0.06	<.0001	0.90
**Foreign patient**	−0.17	−0.73	0.38	0.54	0.84	−0.23	−0.73	0.27	0.37	0.79	−0.08	−0.23	0.07	0.32	0.93
**Patient with 3 or more chronic diseases**	−0.15	−0.36	0.05	0.14	0.86	0.55	0.36	0.74	<.0001	1.73	0.70	0.64	0.77	<.0001	2.02
**Patient with 2 chronic disease**	0.02	−0.18	0.22	0.83	1.02	0.48	0.29	0.67	<.0001	1.62	0.47	0.41	0.54	<.0001	1.61
**Patient with 1 chronic disease**	−0.04	−0.23	0.14	0.64	0.96	0.23	0.05	0.40	0.01	1.26	0.27	0.22	0.33	<.0001	1.32
**2016**	−0.12	−0.27	0.03	0.13	0.89	−0.05	−0.19	0.09	0.50	0.95	0.05	0.00	0.11	0.05	1.06
**2017**	0.01	−0.16	0.17	0.93	1.01	−0.15	−0.30	0.01	0.06	0.86	−0.16	−0.22	− 0.10	<.0001	0.85
**2018**	0.21	0.01	0.40	0.04	1.23	−0.31	−0.49	− 0.13	0.00	0.73	−0.51	− 0.57	−0.44	<.0001	0.60
**Patient whose GP is first year in CHC**	−0.25	−0.81	0.31	0.39	0.78	−0.30	−0.80	0.21	0.25	0.74	−0.08	−0.25	0.10	0.40	0.93
**Patient whose GP is in CHC**	−0.15	−0.31	0.02	0.08	0.86	−0.15	−0.30	0.01	0.06	0.86	−0.02	−0.07	0.04	0.56	0.98
**AICC**	84,029.75										87,321.17				
**BIC**	84,352.87										87,482.73				
**Pearson Chi-Square (value/DF)**	1.01										1.03				
**Vuong statistic**	2.12														

## Discussion

Our findings are consistent with the hypothesis that proximity to primary care emergency services results in significant reductions in inappropriate ED use. Our empirical research consists of two steps. Pursuing Aim 1, we found confirmation of previous results [7] that patients living in an area with a local WiC show an estimated reduction of between 64 and 72% in the probability of inappropriate ED attendance. At the same time, our analysis highlights that both WiCs and CHCs can reduce patient inappropriate emergency care access probability, since for patients whose GP belongs to the local CHC, the magnitude of the reduction in inappropriate ED use is estimated at about 33% (27% in the first year), with an impact apparently greater in influencing the decision to avoid inappropriate ED use completely, but less effective when it comes to modifying the behaviour of the so-called frequent flyers. Turning our attention to the Parma city centre WiC to pursue Aim 2, we found evidence that having a GP belonging to the local CHC reduces the probability of a weekday WiC visit by 11%, with a far more significant impact (43%) during the year the GP first joins. This result suggests not only that CHCs can reduce inappropriate ED attendances but where CHCs and a WiC coexist in the same area, CHCs are able to reduce the probability of visiting the local WiC.

## Conclusions

Demonstrating the CHC’s ability to increase ED appropriateness forces the NHS to reconsider the advisability of leaving other existing organizational solutions unchanged in order to reduce duplication. Our new findings suggest that where CHCs and WiCs coexist in the same area, patients who have a GP in the local CHC are less likely to visit a WiC on a workday, whereas this is not the case during the weekend when CHCs are closed and for eye attendances that are appropriate specialist services provided by the Parma WiC’s additional role as an eye emergency department. In other words, our study suggests it would be advisable to reorganize the local WiC, whilst at the same time pursuing further development of the CHC model.

Given the nature of the administrative available data, our study suffered from two important limitations: the impossibility to analyse deeper the determinants of a patient’s risk of referring to the ED or to the WiC and the lack of any socioeconomic characteristics that could help to better understand the reasons that lead to an inappropriate use of emergency services. Despite these limits, our study can be seen as the first step of a research agenda that aims at providing a complete analysis regarding the capacity of CHCs to induce a more appropriate utilization of hospital emergency departments. In particular, it would be of major interest to evaluate their relative (cost) effectiveness but also focusing on specific group of patients such as children and on specific diseases such as mental health or specific chronic conditions such as diabetes. With the CHC-model development process in its infancy, more promising achievements are expected in the future.

## Data Availability

The ASSR-RER and the Local Health Authority of Parma provided the databases used in this study, based on routine administrative information and carried out in compliance with Emilia-Romagna Regional Authority data processing regulations and the Italian Data Protection Act. Administrative data were anonymized prior to the analysis at the regional statistical office, where each patient is assigned a unique identifier. This identifier does not allow to trace the patient’s identity and other sensitive information but given the confidential nature of our dataset we are not allowed to make it available on the internet. To apply for permissions to obtain access to the raw data please contact segrsst@regione.emilia-romagna.it.

## Abbreviations

CHC: Community Health Centre;
ED: Emergency Department
GP: General Practitioner
WiC: Walk-in-Clinic
LHA: Local Health Authority
NHS: National Health Service
